# Verbesserung des Record Linkage für die Gesundheitsforschung in Deutschland – wie können Defizite behoben werden?

**DOI:** 10.1007/s00103-025-04153-y

**Published:** 2025-11-20

**Authors:** Timm Intemann, Knut Kaulke, Dennis-Kenji Kipker, Vanessa Lettieri, Christoph Stallmann, Carsten O. Schmidt, Martin Bialke, Christopher Hampf, Dana Stahl, Martin Lablans, Klaus Kraywinkel, Sebastian Bartholomäus, Anatol-Fiete Näher, Galina Tremper, Mohamed Lambarki, Stefanie March, Fabian Prasser, Anna Christine Haber, Johannes Drepper, Irene Schlünder, Toralf Kirsten, Iris Pigeot, Ulrich Sax, Benedikt Buchner, Sebastian C. Semler, Wolfgang Ahrens

**Affiliations:** 1 Leibniz-Institut für Präventionsforschung und Epidemiologie – BIPS, Achterstr. 30, 28359 Bremen, Bremen Deutschland; 2 TMF – Technologie- und Methodenplattform für die vernetzte medizinische Forschung e. V., Berlin, Deutschland; 3 Universität Augsburg, Augsburg, Deutschland; 4 Fachbereich Rechtswissenschaft, Universität Bremen, Bremen, Deutschland; 5 Institut für Sozialmedizin und Gesundheitssystemforschung, Otto-von-Guericke-Universität Magdeburg, Magdeburg, Deutschland; 6 Universitätsmedizin Greifswald, Greifswald, Deutschland; 7 Deutsches Krebsforschungszentrum Heidelberg, Heidelberg, Deutschland; 8 Deutsches Konsortium für Translationale Krebsforschung (DKTK), Heidelberg, Deutschland; 9 Medizinische Fakultät Mannheim, Universität Heidelberg, Mannheim, Deutschland; 10 Robert Koch-Institut, Zentrum für Krebsregisterdaten, Berlin, Deutschland; 11 Landeskrebsregister NRW, Bochum, Deutschland; 12 Robert Koch-Institut, Berlin, Deutschland; 13 Institut für Medizinische Informatik, Charité Universitätsmedizin Berlin, Berlin, Deutschland; 14 Universität Potsdam, Hasso Plattner Institute für Digital Engineering, Potsdam, Deutschland; 15 Hochschule Magdeburg-Stendal, Magdeburg, Deutschland; 16 Berlin Institute of Health at Charité – Universitätsmedizin Berlin, Berlin, Deutschland; 17 Universität Leipzig, Leipzig, Deutschland; 18 Hochschule Mittweida, Mittweida, Deutschland; 19 Fachbereich Mathematik und Informatik, Universität Bremen, Bremen, Deutschland; 20 Institut für Medizinische Informatik, Universitätsmedizin Göttingen, Göttingen, Deutschland

**Keywords:** Data Sharing, Daten-Linkage, FAIR-Prinzipien, Forschungsdateninfrastruktur, Unique Identifier, Data sharing, Data linkage, FAIR principles, Research data infrastructure, Unique identifier

## Abstract

Mit der Verknüpfung unterschiedlicher Gesundheitsdaten auf Personenebene (Record Linkage, RL) lassen sich wissenschaftliche Fragestellungen beantworten, die mit einer Datenquelle alleine nicht zu beantworten wären. Die verknüpften Daten entfalten daher ein großes Potenzial für die Gesundheitsforschung, um Prävention, Therapie und Versorgung auf Bevölkerungsebene zu verbessern. Personenbezogene Gesundheitsdaten sind durch strenge Rechtsvorschriften geschützt. Ihre Nutzung bedarf einer Abwägung zwischen dem berechtigten Interesse am Schutz der persönlichen Daten und dem gesundheitlichen Nutzen. Allerdings schränken derzeit Gesetze und ihre Auslegung das RL von Gesundheitsdaten in Deutschland so stark ein, dass ihr Potenzial zur Verbesserung der Gesundheit bisher nur unzureichend genutzt werden kann. Das RL wird in Deutschland insbesondere durch das Fehlen eines Unique Identifier erschwert, der eine fehlerfreie Zusammenführung über verschiedene Datenkörper hinweg ermöglicht. Insgesamt fehlen interoperable Lösungen, um ein umfassendes studien- und datenkörperübergreifendes RL in einer gesicherten Umgebung durchführen zu können.

In diesem Artikel schlagen wir basierend auf dem „White Paper – Verbesserung des Record Linkage für die Gesundheitsforschung in Deutschland“ Lösungen für die personenbezogene Datensatzverknüpfung von unterschiedlichen Datenquellen vor. Unsere Lösungsvorschläge beinhalten u. a. die Etablierung einer Gesundheits-ID und die Schaffung einer dezentral-föderierten Forschungsdateninfrastruktur mit zentralen Komponenten. Auch wenn diese Vorschläge im Einklang mit der Datenschutzgrundverordnung stehen, gibt es im Einzelfall weiteren gesetzlichen Regelungsbedarf.

## Abstract (en)

Linking different health data at the personal level (record linkage, RL) allows answering scientific questions that could otherwise not be answered by a single data source. Linked data therefore offer great potential for health research to improve prevention, treatment, and care at the population level. Personal health data are protected by strict legal regulations. Its use requires balancing legitimate interests in protecting personal data and health benefits. However, current laws and their interpretations in Germany place severe restrictions on health data RL such that its potential for improving health outcomes is still to be leveraged. In Germany, RL is also hindered by the lack of a unique identifier that enables error-free merging across different data sources. Overall, there is a lack of interoperable solutions to perform comprehensive RL across studies and data sources in a secure environment.

In this article, we propose solutions for the linkage of personal health data from different sources based on the *White Paper – Verbesserung des Record Linkage für die Gesundheitsforschung in Deutschland*. Our proposed solutions include, among others, the establishment of a health ID and the creation of a decentralized federated research data infrastructure with central components. Although these proposals are in line with the General Data Protection Regulation, there is a need for further legal regulation in specific cases.

## Hintergrund

Neben Primärdaten, die in Studien gesammelt werden, gibt es in Deutschland einen Schatz an individuellen Gesundheitsdaten, deren Potenzial für die Verbesserung von Prävention, Therapie und Versorgung bei Weitem noch nicht ausgeschöpft ist. Von besonderem Interesse sind hier z. B. Daten der Krankenkassen, der Krankenhäuser, der elektronischen Patientenakte (ePA) und aus Registern wie Krebs- oder Mortalitätsregistern. Abgesehen von Registern werden personenbezogene Informationen in Sekundärdaten nicht primär für die Forschung, sondern für andere Zwecke gespeichert, wie z. B. die Abrechnungsdaten der Krankenkassen. Record Linkage (RL), also die Verknüpfung verschiedener Datenquellen miteinander auf individueller Ebene, ermöglicht, Forschungsfragen zu beantworten, die mit einer Datenquelle alleine nicht beantwortet werden können. Für einen Überblick über die Möglichkeiten für RL-Studien in den nordischen Ländern und die damit zu erzielende Evidenz sei auf [1] verwiesen. Tab. 1 gibt hierzu 3 Beispiele aus den Bereichen Pflege, Impfstoffvalidierung und Umweltepidemiologie. Darüber hinaus könnten z. B. klinische Studiendaten mit Daten zur medizinischen Versorgung oder mit Sozialdaten angereichert werden, was einen ganzheitlichen Ansatz ermöglicht, um z. B. die Wirksamkeit von Arzneimitteln zu ermitteln.

**Tab. 1:** Beispiele von Record-Linkage-Studien aus anderen europäischen Ländern

Forschungsthema	Land	Verknüpfte Datenquellen	Referenz
Risikoschätzung der Pflegebedürftigkeit älterer Menschen zur intelligenten Steuerung von Pflegeangeboten	Dänemark	Melde‑, Bildungs‑, Einkommens‑, Verschreibungs‑, Patienten‑, Pflege- und Mortalitätsregister	[27]
Untersuchung des Nutzens der COVID-19-Impfung bei Jugendlichen	England	Hausarztpraxen, Testlabore und Krankenhäuser sowie Mortalitätsregister	[28]
Untersuchung des Zusammenhangs zwischen Radonexposition und Lungenkrebs	Schweden	Krebs‑, Bevölkerungs- und Mortalitätsregister sowie epidemiologische Primärdaten	[29]

Die Nutzung dieser Daten bedarf einer Abwägung zwischen dem berechtigten Interesse am Schutz der persönlichen Daten der Betroffenen und dem gesundheitlichen Nutzen für die allgemeine Bevölkerung. Eine spezifische Einwilligung des Einzelnen kann in vielen Fällen nicht eingeholt werden. Es ist weder durchführbar noch wissenschaftlich akzeptabel, mehrere Millionen Personen, deren Daten auf individueller Ebene in Sekundärdatenquellen erfasst sind, um eine informierte Einwilligung zur Studienteilnahme zu bitten. Die Einholung einer solchen wäre nicht nur extrem aufwendig, sondern würde auch zu einer stark selektierten Stichprobe führen *(Non-Responder Bias)* und so die Gültigkeit und Verallgemeinerbarkeit der Studienergebnisse einschränken.

Ohne einheitliche, bundesweite gesetzliche Vorgaben sind wichtige Studien nicht durchführbar. Zudem ermöglichen die Informationen in den zu verknüpfenden Datenquellen oft kein exaktes RL. Damit ist das RL nicht nur ein rechtliches, sondern auch ein methodisch-technisches Problem, da in Deutschland, anders als in vielen anderen Ländern, gesundheitsbezogene Daten ohne übergreifende eindeutige Kennung (*Unique Identifier*, UI) gespeichert werden. Je nach Methoden und verfügbaren Identifikatoren ist eine Verknüpfung entweder nicht möglich oder es besteht das Risiko von Verknüpfungsfehlern [2], die ebenfalls die Gültigkeit und Verallgemeinerbarkeit der Forschungsergebnisse einschränken.

In dem „White Paper – Verbesserung des Record Linkage für die Gesundheitsforschung“ [3] werden verschiedene Anwendungsfälle beschrieben, die die technischen und rechtlichen Rahmenbedingungen veranschaulichen, unter denen die Wissenschaft gegenwärtig arbeiten muss, um ein RL verschiedener Datenkörper zu erreichen. Je nach Anwendungsgebiet werden die verwendeten Methoden für ein datenschutzfreundliches RL (*Privacy-Preserving RL*, PPRL) bewertet. Aufbauend auf [4] werden die gewonnenen Erkenntnisse beschrieben. Darauf basierend werden Lösungen vorgeschlagen, die sowohl die Datenschutzgrundverordnung (DSGVO) als auch die wissenschaftlichen Erfordernisse berücksichtigen. In diesem Artikel beschreiben wir den Status quo des RL in Deutschland und stellen nötige Maßnahmen gebündelt dar, um eine Grundlage für die Verbesserung der Gesundheitsforschung in Deutschland zu schaffen. Die Einführung eines UI ist dafür eine zentrale Voraussetzung, die aber alleine das komplexe Problem nicht lösen wird, da zusätzlich auch rechtliche, organisatorische und infrastrukturelle Maßnahmen erforderlich sind. Im Artikel wird zudem das Bild durch eine kurze Betrachtung des Aufwands und Nutzens abgerundet.

## Vorgehen

Um den Status quo des RL für die Gesundheitsforschung zu erfassen, wurden im Rahmen des Projekts NFDI4Health Expert:innen zu einer Reihe von Workshops eingeladen, auf denen Erfahrungen und Meinungen zur Verbesserung des RL in Deutschland ausgetauscht wurden. In insgesamt 10 Anwendungsfällen wurden Erfahrungsberichte aus RL-Projekten unterschiedlicher Bereiche zusammengetragen:

1. Linkage von Primärdaten und Krankenkassendaten,
2. Linkage von Krankenkassen- und Krebsregisterdaten,
3. Linkage der Landeskrebsregister am Beispiel von Nordrhein-Westfalen und Niedersachsen,
4. Linkage am Beispiel der molekularen SARS-CoV-2-Surveillance,
5. Linkage von Daten aus Routinedaten und -proben sowie klinischen Erhebungen innerhalb eines rechtlichen Trägers,
6. Linkage von klinischen Routine- und Studiendaten über mehrere rechtliche Träger hinweg am Beispiel des nationalen Netzwerks Genomische Medizin Lungenkrebs,
7. standortübergreifendes RL im Deutschen Konsortium für Translationale Krebsforschung (DKTK),
8. Spezialfall Treuhandstelle – die Unabhängige Treuhandstelle der Universitätsmedizin Greifswald,
9. standortübergreifendes RL in der Medizininformatik-Initiative (MII),
10. standortübergreifendes RL im Netzwerk Universitätsmedizin (NUM).

Die Anwendungsfälle wurden hinsichtlich rechtlicher Grundlage, Umsetzung des Datenschutzes, verwendeter Identifikatoren, RL-Verfahren und Herausforderungen verglichen. Zudem wurden die rechtlichen Möglichkeiten betrachtet.

## Ergebnisse

### Allgemeine Vorgehensweisen

In den Anwendungsfällen wurde gezeigt, dass Forschende auf unterschiedliche Weise und mit großem Aufwand RL betreiben und dabei mit verschiedenen Herausforderungen konfrontiert werden, um dringende Fragen der Gesundheitsforschung bearbeiten zu können. In den geschilderten Projekten wurden mehrheitlich direkte Identifikatoren (wie z. B. Krankenversichertennummer (KVNR), Name, Vorname) – entweder kodiert oder im Klartext – beim RL eingesetzt. Die meisten Forschungsprojekte verwendeten probabilistische RL-Ansätze. Häufig wurden dabei mit Hashfunktionen^1^ und Bloomfiltern^2^ [5] kodierte Identitätsdaten verwendet. Auftretende Linkage-Unsicherheiten können einen aufwendigen manuellen Abgleich unkodierter Identitätsdaten erfordern. In den untersuchten Projekten wurde die informationelle Gewaltenteilung umgesetzt, indem Forschungsinfrastrukturen in unabhängige Teilstrukturen untergliedert wurden. So können Gesundheitsdaten von den personenidentifizierenden Daten getrennt gehalten werden. Eine Vertrauensstelle administriert und pseudonymisiert dabei die personenidentifizierenden Daten [6, 7].

### Allgemeine Herausforderungen

Allen Anwendungsfällen ist gemein, dass die Fehlerraten für Homonyme (falsch positive Links) und für Synonyme (falsch negative Links) erheblich sein können. Ein Anwendungsfall sticht hierbei mit einer Synonymfehlerquote von 57,6 % heraus – allerdings können auch in anderen Anwendungsfällen Forschungsergebnisse durch Verknüpfungsfehler verzerrt werden. So wurde bei einem RL von Krebsregister- und Krankenkassendaten die Reduktion des Krebsrisikos bei einer Diabetesmedikation mit Sulfonylharnstoffen (im Vergleich zu Metformin) aufgrund von Verknüpfungsfehlern deutlich unterschätzt [8].

In Krebsregistern entsteht durch die manuelle Nachbearbeitung dieser Fehler daher ein erhöhter Personalbedarf [9]. Eine solche Nachbearbeitung ist bei anderen Anwendungen jedoch nicht durchführbar. Die Verwendung der KVNR als direkter Identifikator könnte hier Abhilfe schaffen. Die KVNR steht allerdings für manche Personengruppen (z. B. Selbstzahlende) und in vielen Datenquellen (z. B. bei privaten Krankenversicherungen) nicht immer zur Verfügung.

Daneben stellt auch der Aufwand für die Einholung der erforderlichen Genehmigungen von unterschiedlichen zuständigen Aufsichtsbehörden eine Hürde dar. Dies gilt insbesondere, wenn Datenquellen über mehrere Dateneigner hinweg verteilt vorliegen und unter unterschiedliche rechtliche Regelungen fallen – z. B. bei Landeskrebsregisterdaten oder Abrechnungsdaten aus regionalen oder überregionalen sowie privaten und gesetzlichen Krankenversicherungen.

Insgesamt ist festzuhalten, dass das RL für die Gesundheitsforschung in Deutschland ein aufwendiges Unterfangen darstellt, bei dem die Beteiligten je nach Einsatzbereich, rechtlicher Grundlage, Datenschutzmodell und verfügbaren Identifikatoren aus verschiedenen Optionen wählen müssen. So werden für jeden Anwendungsfall und jedes RL-Projekt einzelfallspezifische Lösungen entwickelt, geprüft, ggf. modifiziert und umgesetzt. In der Praxis ist die Forschung dabei rechtlichen, organisatorischen, personellen und zeitlichen Restriktionen unterworfen. In der Folge werden Forschungsergebnisse aufgrund von Verknüpfungsfehlern verzerrt oder es kommen Studien wegen des Aufwands und der Rechtsunsicherheit erst gar nicht zustande. Es besteht also dringender Verbesserungsbedarf beim RL für die Gesundheitsforschung.

### Rechtliche Betrachtung

Bei Forschungsprojekten unter Verwendung von Sozial- und Gesundheitsdaten sind umfassende datenschutzrechtliche Vorgaben zu beachten. Die Nutzung und das RL unterschiedlicher Datenquellen unterliegen einem komplexen datenschutzrechtlichen Regelungsregime. Dabei bestehen Besonderheiten und Ausnahmen für die Wissenschaft, die spezielle organisatorische Herausforderungen mit sich bringen [10].

Zur Legitimation der Datenverarbeitung, einschließlich des RL, können entweder die datenschutzrechtliche Einwilligung der betroffenen Person oder ein gesetzlicher Erlaubnistatbestand herangezogen werden. Dabei sind je nach Verarbeitungssituation und Datenkategorie unterschiedliche Rechtsgrundlagen als datenschutzrechtliche Legitimationsinstrumente relevant. Gesetzliche Erlaubnistatbestände existieren für Sozial‑, Krebsregister‑, Melderegister‑, Labor- und Versorgungsdaten.

Nach wie vor ist die Einwilligung der betroffenen Person das zentrale Instrument zur Legitimation und Durchführung des RL von personenbezogenen Daten. Andernfalls müssen gesetzliche Erlaubnistatbestände herangezogen werden, die häufig mit hohen Abstimmungsaufwänden verbunden sind. Dies gilt insbesondere dann, wenn landes- oder sektorübergreifende Datenquellen verlinkt werden sollen.

Darüber hinaus ist die Anwendung verschiedener Regelungen auf Europa‑, Bundes- und Landesebene zu RL-Zwecken herausfordernd. Jedoch werden die Bedarfe zur Erleichterung von RL erkannt und zunehmend regulatorisch aufgegriffen: 2 zentrale Beispiele dafür sind das Gesundheitsdatennutzungsgesetz (GDNG)^3^ und der Europäische Raum für Gesundheitsdaten (EHDS)^4^.

## Schlussfolgerung – wie können Defizite behoben werden

Wie in dem White Paper [3] gezeigt wurde, gibt es Herausforderungen, die ein effektives RL unverhältnismäßig erschweren (Infobox 1). Dadurch wird Gesundheitsforschung behindert und teilweise sogar unmöglich gemacht. Im Folgenden machen wir Vorschläge zur Lösung dieser Herausforderungen (Infobox 2). Ein UI kann dabei der Schlüssel für ein sicheres und effizientes RL sein, wenn gleichzeitig folgende Aspekte berücksichtigt werden: Integration des UI in relevanten Datenquellen, Schaffung von Rechtssicherheit bei der Verwendung des UI und bei RL ohne *Informed Consent *(informierte Einwilligung), Aufbau einer zentralen Infrastruktur u. a. zur Vergabe und Verwaltung des UI und nicht zuletzt die gesellschaftliche Akzeptanz als Voraussetzung für die Nutzung von Gesundheitsdaten zu Forschungszwecken. Die Vorschläge richten sich an die Wissenschaftscommunity, an die Verwaltung und insbesondere an die Gesetzgeber. Insgesamt ist ein gemeinsames Vorgehen der Akteure notwendig, um eine Verbesserung der Gesundheitsforschung zu ermöglichen.

### Ausbau und Schaffung von Registern und Bereitstellung von Sekundärdaten zur wissenschaftlichen Nutzung

Eine Voraussetzung für die Durchführung von RL in der Gesundheitsforschung ist die Verfügbarkeit personenbezogener Gesundheitsdaten. Dazu sollten einerseits vorhandene Sekundär- und Registerdaten im Sinne der FAIR-Prinzipien [11] nutzbar gemacht werden. Das heißt, die personenbezogenen Daten müssen auffindbar (*Findable*), zugänglich (*Accessible*), interoperabel (*Interoperable*) und wiederverwendbar (*Reusable*) sein und somit insbesondere ein RL mit anderen Datenquellen ermöglichen – idealerweise mittels eines in den Datenquellen enthaltenen UI. Das betrifft z. B. die Erschließung der ePA oder Informationen zu Impfungen. Zudem sollten weitere personenbezogene Register, wie ein Mortalitätsregister oder medizinische Geburtsregister, bundesweit aufgebaut werden, um das volle Potenzial von RL ausschöpfen zu können. Diese Empfehlungen decken sich mit bereits formulierten Forderungen aus der Wissenschaft und dem Gesundheitswesen [12–14].

### Zentrale Genehmigungsbehörde und Rechtssicherheit

Bei Forschungsvorhaben besteht bezüglich des Datenschutzes häufig eine erhebliche Rechtsunsicherheit, weil die deutschen Datenschutzaufsichtsbehörden keine Genehmigungen erteilen, sondern lediglich bei angenommenen Verstößen einschreiten. Das führt dazu, dass von Ausnahmeklauseln für die Forschung nur selten Gebrauch gemacht wird. Die Anwendung gesetzlicher Ausnahmeregelungen setzt eine Interessenabwägung voraus, die von der verantwortlichen Stelle, die die Daten zur Verfügung stellt, selbst zu treffen ist. Dabei entsteht jedoch das Risiko, dass eine solche Entscheidung im Nachhinein von der verantwortlichen Datenschutzaufsichtsbehörde nicht geteilt wird. Selbst wenn Ausnahmeregelungen im Einzelfall ein Forschungsprojekt ermöglichen könnten, wird diese Möglichkeit nur selten genutzt, weil die Regelungen der DSGVO mit erheblichen Haftungsrisiken einhergehen. Diese werden verständlicherweise im Allgemeinen gescheut. Zudem ist es gerade die Aufgabe von Datenschutzbeauftragten und Rechtsabteilungen, Compliance sicherzustellen. Die sichere Lösung ist daher häufig die Nichtanwendung einer Ausnahmeregelung. Dies erklärt die häufig restriktive Auslegung der Datenschutzregeln. Mit § 6 Abs. 3 GDNG ist nun erstmals eine Vorschrift geschaffen worden, die für die Weiterverarbeitung von Versorgungsdaten u. a. zu Forschungszwecken vorsieht, dass hier die zuständige Datenschutzaufsichtsbehörde einer gemeinsamen Datenverarbeitung etwa im Rahmen von Verbundforschungsvorhaben zustimmen und über diese Zustimmung innerhalb eines Monats entscheiden muss – und damit erstmals auf diesem Weg Rechtssicherheit für die beteiligten Forschenden geschaffen wird. Mit § 5 GDNG wurde eine Regelung zu einer stärkeren Zentralisierung der Datenschutzaufsicht bei Verbundforschungsvorhaben auf der Basis von Versorgungsdaten geschaffen, die allerdings gerade für öffentliche Einrichtungen in stark kooperativ ausgerichteten Projekten noch keine Verbesserung bringt.

Eine Regelung wie § 6 Abs. 3 GDNG hat das Potenzial, die Möglichkeiten für ein RL im Rahmen von Verbundforschungsvorhaben rechtssicher zu erweitern. Wenn Entscheidungen über Ausnahmeregelungen zum Einwilligungserfordernis staatlich geprüft und klagefähig entschieden werden, kann sichergestellt werden, dass hier eine Kasuistik entsteht, aus der sich eine größere Rechts- und Planungssicherheit ergibt. Auch gegenüber Betroffenen ist diese Lösung vorteilhaft, weil sie Transparenz herstellt und Betroffenenrechte durch ein Anfechtungsrecht der behördlichen Entscheidung ermöglicht.

In Frankreich existiert mit der *Commission Nationale de l’Informatique et des Libertés* eine solche federführende Aufsichtsbehörde, die eine Genehmigungskompetenz besitzt. Es wäre wünschenswert, auch in Deutschland eine solche Institution für den Bereich der Gesundheitsforschung einzurichten. Diese könnte dafür sorgen, Rechtssicherheit bei der Nutzung von Forschungsklauseln zur einwilligungsfreien Datennutzung sowie bei der Verwendung eines UI herzustellen und haftungsrechtliche Risiken zu vermeiden. Ansätze hierzu finden sich z. B. beim EHDS^5^. Die derzeitig bestehende Rechtsunsicherheit führt zudem dazu, dass ein notwendiger Diskurs zur rechtspolitischen Überarbeitung der gesetzlichen Ausnahmeregelungen nicht entsteht, weil die jeweiligen Forschungsprojekte gar nicht erst zustande kommen. Auch dieses Problem könnte durch eine solche staatliche Stelle gelöst werden.

### Regelungsbedarf bei RL ohne Informed Consent

Bei vielen Forschungsvorhaben wird die informierte Einwilligung eingeholt, bevor ein RL durchgeführt wird. Diese spezifische persönliche Einwilligung legt bestimmte RL-Möglichkeiten fest, die sich später nicht mehr ändern lassen, ohne die Teilnehmenden erneut zu kontaktieren. Aufgrund des Aufwands wird davon in der Regel abgesehen. Da das Datenschutzrecht bislang abgesehen vom sog. Broad Consent kaum Möglichkeiten eröffnet, Einwilligungsdokumente so zu verfassen, dass sie möglichst umfassende RL-Szenarien abdecken, entstehen hierdurch notwendigerweise Lücken im Hinblick auf das RL in zukünftigen Forschungsprojekten.

In der Situation, in der Datensätze, die ohne Einwilligung erhoben wurden und somit kein Kontakt zu den Personen hergestellt wurde, mit Datensätzen, die nur mit Einwilligung verwendet werden dürfen, verknüpft werden sollen, besteht Regelungsbedarf. Es müsste in der entsprechenden gesetzlichen Grundlage ein zukünftiges RL mitgedacht werden, das über die initiale gesetzliche Zweckgebundenheit hinausgeht. So sind zurzeit Registerdaten nicht mit Biobankdaten standortübergreifend verknüpfbar, wenn weder die Einwilligung der Bioprobenspender:innen noch das entsprechende Registergesetz eine solche Verlinkung vorsieht. § 6 Abs. 1 GDNG schafft hier neue Möglichkeiten zum RL, soweit personenbezogene Daten z. B. für die medizinische Diagnostik verarbeitet werden, indem eine explizite zweckändernde Weiterverarbeitung auch zu Zwecken der medizinischen Forschung ermöglicht wird. Für Daten aus der ärztlichen Behandlung ist es zudem wichtig, dass gesetzliche Regelungen so formuliert werden, dass sie sicher als Offenbarungsbefugnis nach § 203 StGB ausgelegt werden können. Dies gilt derzeit noch nicht für viele Ausnahmeklauseln zum Einwilligungserfordernis, die für Forschungszwecke anwendbar sind.

### UI

Record Linkage-Verfahren ohne einen UI sind aufwendig und fehleranfällig. Daher bleibt der Ruf nach einer solchen eindeutigen persönlichen Kennung für das RL berechtigt [12, 14–16]. Wünschenswerte Eigenschaften eines UI wurden bereits zusammengetragen [17]. Von den existierenden Identifikationsnummern (IDs) in Deutschland kommen für die Ableitung eines UI infrage:

- die Sozial- bzw. Rentenversicherungsnummer (SVNR/RVNR),
- die daraus abgeleitete KVNR,
- die elektronische Identität aus dem Personalausweis (eID) und
- die Steuer-ID (nach § 139b Abgabeordnung), die nahezu alle Personen abdeckt und die in 51 Behörden, Datenbanken und Verzeichnissen geführt wird, womit ihr die Rolle einer Bürger-ID zukommen kann.

Aus diesen Identifikatoren könnte ein geschütztes bereichsspezifisches Pseudonym, eine sogenannte Gesundheits-ID (oder „Forschungspseudonym“) erzeugt werden. Eine solche neu zu schaffende, versorgungsnahe Gesundheits-ID könnte das Problem der Verknüpfungsfehler lösen, ohne dass ein RL mit personenidentifizierenden Daten aus anderen Bereichen erforderlich ist. Zudem wäre damit das RL deutlich effizienter.

Zur Erarbeitung einer Gesundheits-ID schlagen wir die Einrichtung einer interdisziplinären Kommission unter Führung des Bundesministeriums für Gesundheit vor. Diese sollte Erfahrungen aus anderen europäischen Ländern und Vorgaben für die eID der EU-Bürger:innen gemäß der eIDAS-Verordnung^6^ berücksichtigen. Verwaltungswissenschafts‑, Sicherheits- und Domänenexpertise der Sozialversicherungen, der Gesundheitsforschung sowie der Versorgung sollten einbezogen werden (für Details siehe [15]).

### Infrastruktur und Servicestruktur für RL mit zentralen Komponenten

Die Datenerfassung und -speicherung in der Gesundheitsversorgung sowie in der klinischen und epidemiologischen Forschung erfolgen dezentral. Um diese dezentralen Strukturen sinnvoll zu verbinden und sowohl eine doppelte als auch zentrale Datenhaltung zu vermeiden, sollte eine dezentrale Forschungsdateninfrastruktur (FDI) aufgebaut werden – einen solchen Ansatz verfolgt auch das GDNG mit einem öffentlichen Metadaten-Katalog, der nicht die eigentlichen Datensätze, sondern u. a. die Informationen über Datenhalter und Lokalisierung der Forschungsdaten enthält.

Um alle relevanten Gesundheitsdaten sicher, umfassend und effektiv für die Forschung zu nutzen, bedarf es einer FDI, die ein fehlerfreies RL und ein Identitätsmanagement umsetzt und dabei mittels Pseudonymisierungstechniken höchste Datenschutzanforderungen erfüllt. Dabei sollten Daten anlassbezogen für die Forschung verknüpft werden können. Hierfür erscheint es sinnvoll, bestehende Strukturen der Datenhalter und FDI einzubinden, wie z. B. die Datenintegrationszentren (DIZ) der Universitätsmedizin für die MII und das NUM, die Local Data Hubs der NFDI4Health^7^, das Forschungsdatenzentrum Gesundheit [18], die onkologischen Brückenköpfe des DKTK [19], das Krebs-Forschungsdatenzentrum onkoFDZ^8^ sowie die Krebsregister.

Die DIZ und Brückenköpfe repräsentieren bereits existierende dezentrale Infrastrukturen, die für die sekundäre Datennutzung konzipiert sind. Sie ermöglichen an ihren jeweiligen Standorten den Zugang zu Gesundheitsdaten für Forschungszwecke. In ihrer Funktion agieren sie als lokale Data Hubs innerhalb einer föderierten FDI. Die gleiche Idee verfolgt die NFDI4Health mit der Einrichtung von *Local Data Hubs *für alle forschungsrelevanten personenbezogenen Gesundheitsdaten, weswegen eine intensivere Zusammenarbeit der Initiativen notwendig ist, um Doppelstrukturen zu vermeiden.

Für eine effektive dezentral-föderierte FDI zur Erreichung eines umfassenden RL sind zentrale Regelungen zu Governance und Koordination sowie zentrale Komponenten für das RL unverzichtbar:

- Um der Notwendigkeit eines UI beim RL von personenbezogenen Gesundheitsdaten Rechnung zu tragen, sollten zentrale Instanzen die Aufgabe zur Vergabe und Verwaltung von Identifikatoren übernehmen. Insbesondere sollte eine zentrale Vertrauensstelle die bereichsspezifische Gesundheits-ID zuordnen und verwalten.
- Zusätzlich bedarf es einer Vereinheitlichung von Regeln für das RL und die Pseudonymisierung, um höchsten Ansprüchen an den Datenschutz zu genügen, damit in einer sicheren Umgebung ein exaktes RL unter Verwendung der Gesundheits-ID erfolgen kann. Da die Verknüpfung der Identitäten und die Pseudonymzuweisung eng miteinander verbunden sind, muss eine zentrale Instanz für beide Vorgänge grundlegende Vorgaben festlegen. Mit der *Federated Trusted Third Party* [20] und dem Pseudonymisierungsdienst *Mainzellisten-PSD* [21] gibt es hierzu bereits Vorarbeiten.
- Über die ePA sollte ein Zugang zu institutionsübergreifenden Versorgungsdaten für Forschende gemäß § 363 SGB V geschaffen werden. Die ePA dient dann nicht nur als zentraler Zugriffspunkt für Patient:innen, sondern ermöglicht auch die Verwaltung persönlicher Einwilligungen. Dafür sollte eine zentrale Instanz zur Verwaltung von Einwilligungen etabliert werden.
- Die Einführung eines zentralen Transparenzportals zur Information der Bürger:innen über die Datennutzung könnte die Akzeptanz in der Bevölkerung erhöhen.
- Die dezentralen Daten, die für das RL zur Verfügung stehen, sollten über ein zentrales Anfrageportal zugänglich gemacht werden. Ein solches Portal ermöglicht Forschenden, Daten und Datenzugänge zu überblicken und RL-Anfragen zu stellen. Dieses Portal sollte einen Zugang zu relevanten Informationen bieten (inkl. Übersicht über Datenbestände und vertragliche Regelungen). Mit dem Forschungsdatenportal für Gesundheit (FDPG)^9^ der MII, dem Föderierten Explorer des DKTK^10^ und dem Sample Locator des German Biobank Node [22] existieren hierzu bereits Vorarbeiten. Darüber hinaus werden über den German Central Health Study Hub der NFDI4Health bereits Daten epidemiologischer und klinischer Studien, Sekundär- und Registerdaten sowie Public-Health-Daten auffindbar gemacht.^11^ Ein kurzfristiges Ziel muss daher eine Verzahnung der Services sein, um Parallelstrukturen zu vermeiden.
- Der EHDS „fördert die Nutzung von Gesundheitsdaten für eine bessere medizinische Versorgung, für Forschung, Innovation und Politikgestaltung und ermöglicht es der EU, das Potenzial von Austausch, Nutzung und Weiterverwendung von Gesundheitsdaten unter gesicherten Bedingungen voll auszuschöpfen“^12^. Um den Anschluss der nationalen FDI an den EHDS zu ermöglichen, ist es wichtig, eine Hub-Funktion zu etablieren, die ermöglicht, Daten mit anderen Institutionen des EHDS zu teilen.

### Kompetenzförderung und Verfahrenssicherheit bei Pseudonymisierung und Linkage

Zur Bewältigung der methodisch-technischen Herausforderungen beim RL ist es zudem ratsam, Know-how bereitzustellen. Wichtig ist es hierbei, den Wissenstransfer zu Pseudonymisierungs- und RL-Verfahren sowie zur Verwendung eines UI zu fördern. Es wird empfohlen, dass Fachworkshops unterstützt werden. Diese Workshops sollten praktische Verfahrensweisen, verfügbare Technologien und Werkzeuge sowie kontinuierliche Weiterentwicklungen vermitteln (z. B. zu Secure Multi-Party Computation^13^ [23, 24]). Die Relevanz und gleichzeitig Herausforderung in diesem Zusammenhang werden durch die Regelung in § 7 GDNG verdeutlicht, die neue Geheimhaltungspflichten auch im Sinne zusätzlicher Datensicherheit für Forscher:innen enthält, deren Verletzung auch strafbewehrt sein kann.

### Gesellschaftliche Akzeptanz

Die Förderung des gesellschaftlichen Vertrauens in Institutionen, Personen und Prozesse, die für die Auswertung von Gesundheitsdaten verantwortlich sind, stellt eine zentrale Voraussetzung für die Einführung eines UI für die Gesundheitsforschung dar. Das Vertrauen der Bürger:innen in die Gesundheitsforschung ist entscheidend dafür, dass Gesundheitsdaten mit anderen Daten zu Forschungszwecken verknüpft werden können. Daher ist es erforderlich, eine umfassende gesellschaftliche Diskussion über die Vorteile der datenintensiven Gesundheitsforschung zu führen, die 1) neue Modelle der Einwilligung, wie das reine Widerspruchsrecht (Opt-out), und 2) eine stärkere Partizipation der Bevölkerung einschließt.

Zusätzlich sollte ein Transparenzportal über Forschungsvorhaben und Betroffenenrechte informieren und Informationskampagnen unterstützen. Auch hierfür existieren mit dem FDPG der MII und mit dem Citizen-Science-Projekten^14^ der NFDI4Health Vorarbeiten.

## Aufwand und Nutzen

Bei den Aufwänden zu einer umfassenden, bundesweiten RL-Infrastruktur für die deutsche Gesundheitsforschung, die zentrale Komponenten zur Vergabe eines UI und zur Pseudonymisierung umfasst, müssen sowohl Initialaufwände als auch der Aufwand für den laufenden Betrieb berücksichtigt werden. Neben technischen Maßnahmen stehen dabei organisatorische, rechtliche und personelle Aspekte im Fokus. Dem steht ein breiter Nutzen gegenüber – für Forschung, politische Steuerung und Patient:innenversorgung.

Die Bereitstellung einer datenschutzkonformen RL-Infrastruktur erfordert Entwicklungsarbeit zur Anpassung bestehender Tools (aus NFDI4Health, MII und NUM, wie beispielweise Study Hub, FDPG, Mainzelliste^15^, E‑PIX^16^) sowie deren Ausweitung und Integration in die bestehende IT-Landschaft. Der technische Aufwand umfasst Planung, Entwicklung, Testung, Schnittstellenprogrammierung und Qualitätssicherung. Hier muss mit hohen Aufwänden gerechnet werden. Für eine föderierte RL-Infrastruktur sind Rechenzentren, Netzwerkinfrastrukturen, Authentifizierungsprozesse und Sicherheitsvorkehrungen aufzubauen. Zusätzlich müssen Schulungsmaterialien und Helpdesks bereitgestellt werden. Es sind Projektteams aus IT, Recht, Datenschutz, Ethik und Governance erforderlich. Die Entwicklung rechtlich belastbarer Rahmenbedingungen setzt intensive Abstimmungen mit Datenschutzbehörden, Ethikkommissionen und politischen Entscheidungsträgern voraus. Der Aufwand für juristische Begleitung, Richtlinienerstellung und Etablierung klarer Zuständigkeiten ist erheblich, könnte jedoch durch Angleichung der länderspezifischen Regelungen erheblich reduziert werden.

Eine RL-Infrastruktur erfordert dauerhaft Fachpersonal für RL-Prozesse, technische Betreuung, Datenpflege und Kommunikation mit Datengebern. Aufwände entstehen für Softwarepflege, Updates, IT-Support, Monitoring und Sicherheitsvorkehrungen. Dazu gehören auch regelmäßige Weiterentwicklungen der RL-Methodik. Das Vertragsmanagement, Genehmigungsverfahren und die Governance werden dauerhaft Ressourcen binden. Sowohl unter ökonomischen als auch unter sicherheitstechnischen Aspekten sollte eine zentrale RL-Infrastruktur angestrebt werden.

Demgegenüber könnte der Effizienzgewinn immens sein, da eine RL-Infrastruktur eine ressourcenschonende Wiederverwendung vorhandener Daten ermöglicht und Redundanzen verhindert. Forschungsprozesse werden beschleunigt, Ressourcen gezielter eingesetzt und Entscheidungen fundierter getroffen. RL schafft die Basis u. a. für gezieltere Interventionen bei chronischen Erkrankungen, frühzeitige Risikoerkennung und patientenzentrierte Versorgungspfade. Die systematische Verknüpfung verteilter Datenquellen über einen UI steigert die Validität und Informationsdichte von Forschungsdaten. Dies ermöglicht eine Ausweitung an zu beantwortenden Fragestellungen und eine höhere Aussagekraft der Ergebnisse. Mit einem umfassenden RL können individuelle Krankheitsverläufe über Jahre nachverfolgt und mit externen Datenquellen verknüpft werden. Gerade für epidemiologische und gesundheitsökonomische Studien wäre dies ein entscheidender Fortschritt. Standardisierte Datenzugänge und automatisierte Pseudonymisierung verringern Hürden für die Teilnahme an Forschungsprojekten. Eine effektive Infrastruktur schafft Anreize für interdisziplinäre und standortübergreifende Forschungsvorhaben, wie Beispiele aus dem europäischen Ausland zeigen (Tab. 1): Diese Beispiele veranschaulichen die Möglichkeiten einer effektiven RL-Infrastruktur. Sie zeigen, dass Impfkampagnen noch während ihrer Laufzeit umfassend evaluiert und Pflegeangebote signifikant verbessert werden können. Aufgrund der größeren Bevölkerung in Deutschland wären zudem Analysen z. B. zu seltenen Expositionen oder Krankheiten möglich, die in den nordischen Ländern keine ausreichende Aussagekraft erreichen können. So können Patient:innen von evidenzbasierten Entscheidungen und frühzeitiger Risikoidentifikation profitieren. Eine leistungsfähige RL-Infrastruktur trägt zudem zur internationalen Wettbewerbsfähigkeit deutscher Forschungsinstitutionen bei.

Insgesamt erfordert die Einführung einer datenschutzkonformen RL-Infrastruktur in Deutschland einen signifikanten Aufwand. Demgegenüber steht ein breiter Nutzen für Forschung, Versorgung, Ärzt:innen und Patient:innen. Bei der Beurteilung des Aufwands und des Nutzens muss bedacht werden, dass auf vorhandene Infrastrukturen aufgebaut werden kann (u. a. von NFDI4Health, MII, NUM und Forschungsdatenzentrum Gesundheit) und bereits gesetzliche Regelungen verabschiedet wurden, die nicht nur die Basis für eine umfassende RL-Infrastruktur legen, sondern auch entsprechende Infrastrukturanpassungen nötig machen werden (GDNG und EHDS).

## Fazit und Ausblick

Abschließend ist festzuhalten, dass das RL bisher für die Gesundheitsforschung in Deutschland ein aufwendiges und rechtlich unsicheres Unterfangen darstellt. Aufgrund des Fehlens eines UI und des hohen administrativen, rechtlichen und organisatorischen Aufwands bei der Umsetzung kommt es zu hohen Umsetzungskosten, Verknüpfungsfehlern und geringer Nutzung der verfügbaren Daten. Das volle Potenzial vorliegender Daten wird nicht ausgeschöpft. Rechtliche Unsicherheiten führen sogar dazu, dass RL-Vorhaben erst gar nicht durchgeführt werden, sodass wichtige medizinische Fragen unbeantwortet bleiben. Das Nutzenpotenzial rechtfertigt unseres Erachtens den Aufwand, zumal der größte Teil des Aufwands nur einmalig entsteht, und zwar bei der Implementierung eines effektiven RL-Systems. Dieser kann wiederum durch eine sinnvolle Nutzung der in der Entstehung befindlichen Infrastrukturen bedingt durch MII, NUM, NFDI4Health und EHDS reduziert werden.

Unsere Vorschläge sollen zur umfassenden und dringend nötigen Verbesserung des RL für die Gesundheitsforschung beitragen. Sie sind als Forderungen an den Gesetzgeber zu verstehen, eine bessere Grundlage für das RL zu schaffen. Die Wissenschaftsgemeinschaft hat es sehr begrüßt, dass der Gesetzgeber im GDNG mit dem RL zwischen Krankenkassendaten und Krebsregisterdaten sowie der Einführung eines Forschungspseudonyms bereits einige ihrer Vorschläge aufgegriffen hat [25, 26]. Ein bundesweites integratives RL-Konzept, das weitere Gesundheitsdatenquellen einbezieht und Rechtssicherheit gewährleistet, bleibt jedoch unerlässlich. Insbesondere wird es notwendig sein, die Umsetzung von nationalem GDNG und europäischem EHDS in Einklang zu bringen, um nicht nur Umsetzungsaufwände zu vermeiden, sondern tatsächlich zu einem ersten gemeinsamen EU-Datenraum zu gelangen.

### Infobox 1 Herausforderungen beim Record Linkage (aus [3], modifiziert, Lizenz: CC BY-NC 4.0)

- Je nach Verarbeitungssituation und Datenkategorie unterschiedliche Rechtsgrundlagen
- Fragmentierte aufsichts- und fachbehördliche Kontrollstrukturen und damit erheblicher Mehraufwand für Behörden und insbesondere die Wissenschaft
- Föderale Zersplitterung der Datenschutzaufsicht bzw. der zuständigen Aufsichtsbehörden und unterschiedliche Auslegung gesetzlicher Vorgaben
- Hoher bürokratischer und zeitlicher Aufwand für unterschiedliche Genehmigungsverfahren bei Nutzung von Daten mehrerer Datenhalter aus mehreren Bundesländern
- Record Linkage ohne Unique Identifier führt zu Verknüpfungsfehlern und Datenverlust
- *Insgesamt führen diese Beschränkungen dazu, dass nur (Bruch‑)Teile der bestehenden Daten verknüpft und für die Gesundheitsforschung genutzt werden können. Das heißt, Forschung wird verhindert oder auf unzureichende Ergebnisse mit eingeschränkter Aussagekraft beschränkt*

### Infobox 2 Empfehlungen zur Verbesserung des Record Linkage (aus [3], modifiziert, Lizenz: CC BY-NC 4.0)

- Ausbau und Schaffung von Registern und Bereitstellung von Sekundärdaten zur wissenschaftlichen Nutzung
- Einführung einer federführenden Aufsichtsbehörde, inkl. Genehmigungskompetenz, zur Schaffung von Rechtssicherheit
- Einführung eines Unique Identifier und Etablierung von bereichsspezifischen Pseudonymen
- Etablierung einer dezentral-föderierten Forschungsdateninfrastruktur mit zentralen Komponenten für das Record Linkage u. a. zur
  - Vergabe von bundeseinheitlichen Identifikatoren
  - Pseudonymisierung und standardisierten Durchführung des Record Linkage
  - Zuordnung von bereichsspezifischen Pseudonymen (zentrale Vertrauensstelle)
  - Verwaltung von Einwilligungen
  - Beantwortung und Bearbeitung von Anfragen und Anträgen
- Schaffung von Anknüpfungspunkten an den NFDI4Health German Central Health Study Hub, das Forschungsdatenportal für Gesundheit (FDPG) der MII sowie an den Europäischen Raum für Gesundheitsdaten (EHDS)

## Footnotes

1 Durch eine Hashfunktion wird eine Zeichenfolge (z. B. Namen) in einen eindeutigen numerischen Wert fester Länge (z. B. 10-stellige Zahl) umgewandelt.

2 Bloomfilter nutzen Hashfunktionen, um Identitätsdaten in Ketten von Nullen und Einsen zu überführen. Sie sind ähnlichkeitserhaltend und können somit für fehlertolerantes RL eingesetzt werden.

3 https://www.recht.bund.de/bgbl/1/2024/102/VO.html, Zugriff: 14.11.2024.

4 https://eur-lex.europa.eu/eli/reg/2025/327/oj, Zugriff 27.04.2025.

5 https://health.ec.europa.eu/ehealth-digital-health-and-care/european-health-data-space_de, Zugriff: 14.11.2024.

6 Verordnung (EU) Nr. 910/2014 des Europäischen Parlaments und des Rates vom 23. Juli 2014 über elektronische Identifizierung und Vertrauensdienste für elektronische Transaktionen im Binnenmarkt und zur Aufhebung der Richtlinie 1999/93/EG.

7 https://www.nfdi4health.de/en/service/local-data-hub.html, Zugriff 14.11.2024.

8 https://www.bundesgesundheitsministerium.de/ministerium/ressortforschung/handlungsfelder/forschungsschwerpunkte/krebsregisterdaten/onkofdz.html, Zugriff: 14.11.2024.

9 https://forschen-fuer-gesundheit.de, Zugriff: 14.11.2024.

10 https://dktk.dkfz.de/forschung/Plattformen-und-Technologie-Netzwerke/klinische-plattformen/ccp-explorer, Zugriff 05.11.2024.

11 https://csh.nfdi4health.de, Zugriff: 14.11.2024.

12 https://health.ec.europa.eu/ehealth-digital-health-and-care/european-health-data-space_de, Zugriff: 14.11.2024.

13 Secure-Multi-Party-Computation-basierte Verfahren bieten mathematisch beweisbare Sicherheit, dass die personenidentifizierenden Merkmale verborgen bleiben.

14 https://www.nfdi4health.de/en/community-eng/participation.html, Zugriff: 18.07.2025.

15 https://www.unimedizin-mainz.de/imbei/informatik/ag-verbundforschung/mainzelliste.html, Zugriff: 07.07.2025.

16 https://www.ths-greifswald.de/en/researchers-general-public/e-pix/, Zugriff: 07.07.2025.
